# Dual-Labeled Near-Infrared/^99m^Tc Imaging Probes Using PAMAM-Coated Silica Nanoparticles for the Imaging of HER2-Expressing Cancer Cells

**DOI:** 10.3390/ijms17071086

**Published:** 2016-07-07

**Authors:** Haruka Yamaguchi, Makoto Tsuchimochi, Kazuhide Hayama, Tomoyuki Kawase, Norio Tsubokawa

**Affiliations:** 1 Quantitative Diagnostic Imaging Program, Graduate School of Life Dentistry at Niigata, The Nippon Dental University, Niigata 951-8580, Japan; harukay@ngt.ndu.ac.jp; 2 Department of Oral and Maxillofacial Radiology, The Nippon Dental University School of Life Dentistry at Niigata, Niigata 951-8580, Japan; hayama@ngt.ndu.ac.jp; 3 Advanced Research Center, The Nippon Dental University School of Life Dentistry at Niigata, Niigata 951-8580, Japan; kawase@dent.niigata-u.ac.jp (T.K.); ntsuboka@eng.niigata-u.ac.jp (N.T.); 4 Division of Oral Bioengineering, Institute of Medicine and Dentistry, Niigata University, Niigata 951-8514, Japan; 5 Faculty of Engineering, Niigata University, Niigata 950-2181, Japan

**Keywords:** dual-imaging probe, silica nanoparticles, HER2, ^99m^Tc, NIR, ICG, SK-BR3, MDA-MB231, athymic mice, xenograft

## Abstract

We sought to develop dual-modality imaging probes using functionalized silica nanoparticles to target human epidermal growth factor receptor 2 (HER2)-overexpressing breast cancer cells and achieve efficient target imaging of HER2-expressing tumors. Polyamidoamine-based functionalized silica nanoparticles (PCSNs) for multimodal imaging were synthesized with near-infrared (NIR) fluorescence (indocyanine green (ICG)) and technetium-99m (^99m^Tc) radioactivity. Anti-HER2 antibodies were bound to the labeled PCSNs. These dual-imaging probes were tested to image HER2-overexpressing breast carcinoma cells. In vivo imaging was also examined in breast tumor xenograft models in mice. SK-BR3 (HER2 positive) cells were imaged with stronger NIR fluorescent signals than that in MDA-MB231 (HER2 negative) cells. The increased radioactivity of the SK-BR3 cells was also confirmed by phosphor imaging. NIR images showed strong fluorescent signals in the SK-BR3 tumor model compared to muscle tissues and the MDA-MB231 tumor model. Automatic well counting results showed increased radioactivity in the SK-BR3 xenograft tumors. We developed functionalized silica nanoparticles loaded with ^99m^Tc and ICG for the targeting and imaging of HER2-expressing cells. The dual-imaging probes efficiently imaged HER2-overexpressing cells. Although further studies are needed to produce efficient isotope labeling, the results suggest that the multifunctional silica nanoparticles are a promising vehicle for imaging specific components of the cell membrane in a dual-modality manner.

## 1. Introduction

In recent years, cancer therapy has substantially changed with the development of molecularly targeted drugs. It is now expected that clinical molecular imaging can provide important information regarding drug responses, as well as precise diagnoses and prognoses of diseases. Furthermore, a newly emerging concept, theranostics (and more specifically theranostic nanomedicine), which combines diagnostics and therapy, has attracted the attention of researchers and clinicians. Although introduction of these new trends and directions into the clinic is slow, researchers have strived to develop nanoparticle delivery systems for drugs and imaging agents in experimental settings [1,2]. Nanoparticles are classified into two major categories; organic and inorganic nanoparticles [3]. The former includes liposomes, polymeric nanoparticles, micelles, dendrimers, and solid lipid nanoparticles, and the latter includes iron oxide nanoparticles, gold nanoparticles, semiconductor nanocrystals, ceramic nanoparticles, and carbon nanotubes. Nanoscale particle technologies have progressed in recent years [2,4,5]. We previously reported the use of functionalized silica nanoparticle probes-polyamidoamine (PAMAM) dendrimer coated silica nanoparticles (PCSNs) loaded with technetium-99m (^99m^Tc) and indocyanine green (ICG)—to image sentinel lymph nodes in an experimental animal model [6]. Multifunctional nanoparticles including multimodal imaging probes have the potential to create revolutionary changes in medicine [1,7]. The most attractive feature of multifunctional nanoparticles is their ability to target specific cells or sites and to deliver agents to these sites. Combining nanotechnology and biomedical engineering presents a challenge and an opportunity to create nanocarriers with maximum versatility. These combinatorial approaches are classified as liposome-based, polymer-based, or inorganic-based nanosystems [8]. PCSNs are part of the inorganic-based combinatorial nanosystem and may be able to target directly. In this study, we chose human epidermal growth factor receptor 2 (HER2) as the target. HER2 is a transmembrane receptor that responds to signals for promoting and controlling cell proliferation in normal cells. Over-expression or amplification of the HER2 gene occurs in 15%–20% of the breast cancers and this is related to a high recurrence rate of the disease and a poor prognosis [9,10]. Currently, immunohistochemistry and fluorescence in situ hybridization are used to identify HER2-positive breast cancer and to decide whether a patient is eligible for HER2-targeted therapy (e.g., inhibition of HER2-triggered signal transduction by humanized antibodies, small molecules targeting catalytic activity of receptors in clinical trials, and new anti-HER2 strategies) [11]. However, these tests are invasive and restricted to part of the tumor. Biopsies of a small portion of the tumor may not provide a representative HER2 expression status found in the tumor as a whole because of intra-tumor heterogeneity [12]. In vivo imaging of HER2 expression would enable accurate detection of the expression as a whole. New molecular imaging nano-technologies are promising for the development of non-invasive in vivo imaging of HER2 and other disease-specific molecules [13,14]. Our PCSNs could serve as a versatile platform for molecular imaging and therapy. In this study, we investigated the efficacy of functionalized silica nanoparticles with respect to imaging HER2-expressing cells and xenografted tumors using dual-imaging that utilized ^99m^Tc and ICG.

## 2. Results

### 2.1. In Vitro

#### 2.1.1. Imaging of Human Epidermal Growth Factor Receptor 2 (HER2)-Overexpressing Cells (SK-BR3) Using Fluorescent Probes

**Figure 1:**
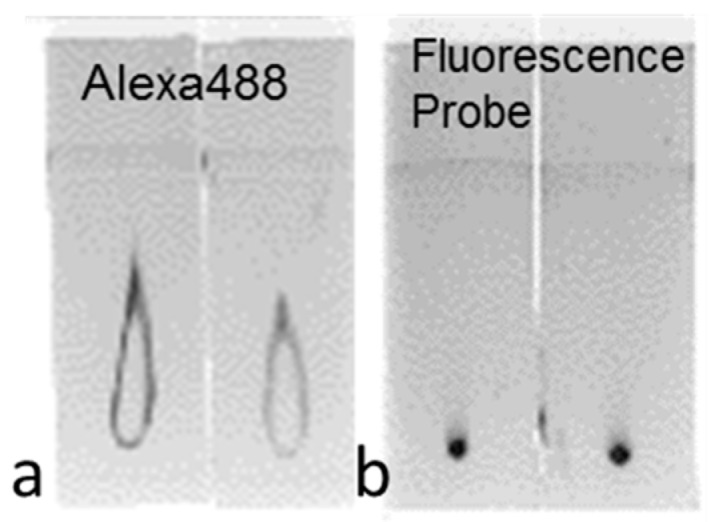
Although only the fluorescent tags ran on the TLC plates (**a**), fluorescent tags of Alexa488 were not separated from polyamidoamine-based functionalized silica nanoparticles (PCSNs) conjugated droplets on thin-layer chromatography (TLC) plates (**b**).

**Figure 2:**
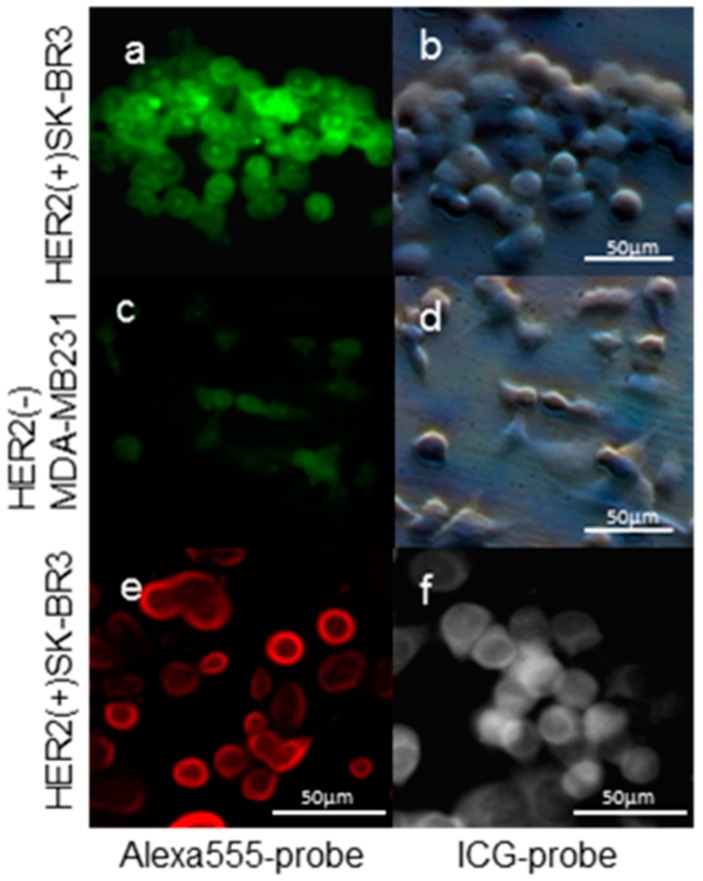
Fluorescence image of human epidermal growth factor receptor 2 (HER2)-targeted PCSNs. SK-BR3 cells (**a**,**b**) exhibited stronger fluorescent signals of Alexa488 than MDA-MB231 cells (**c**,**d**) as demonstrated by the addition of dye-conjugated HER2 targeted PCSNs to the conditioned medium. Alexa 555-probe in SK-BR3 cells (**e**); and indocyanine green (ICG)-probe in SK-BR3 cells (**f**).

**Figure 3:**
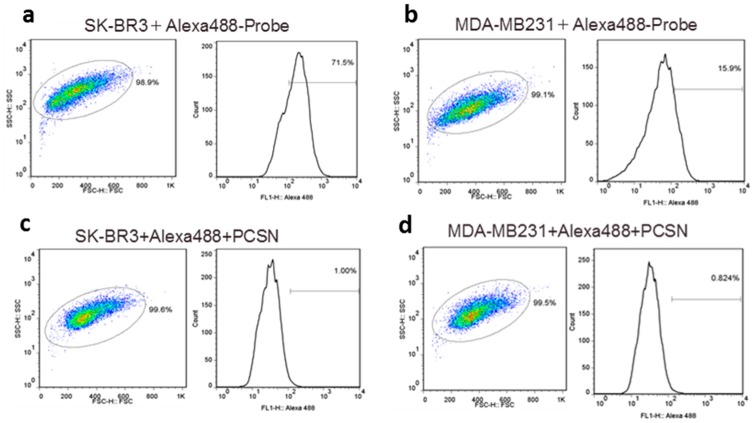
Flow cytometry analysis of HER2-targeted PCSNs. HER2-targeted PCSNs reveals a higher percentage of fluorescent cells in the SK-BR3 cell population (**a**) than in the MDA-MB231 cell population (**b**) and in the non-targeted PCSN controls composed of SK-BR3 cells (**c**) and in the MDA-MB231 cells (**d**). Each cell is represented by a dot and the red/yellow/green/blue hotspots indicate increasing cell populations.

**Figure 4:**
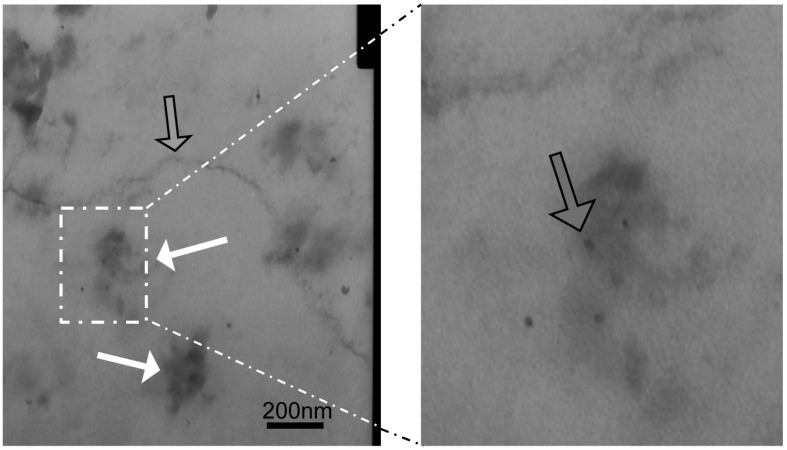
Localization of HER2 targeted PCSNs. TEM image of TEM micrographs showing HER2 targeted PCSNs localization (white arrow) on the surface of the cell membrane (grey arrow) of SK-BR3 cells (**left**). The portion surrounded by a rectangular frame is magnified (**right**).

Fluorescent tags of Alexa 488 were not separated from PCSN-conjugated droplets on thin-layer chromatography (TLC) plates (Figure 1); therefore, the attachment of the fluorescent dye to the PCSNs was confirmed. After exposure to the PCSN probes, SK-BR3 cells had stronger fluorescent signals than the MDA-MB231 cells (Figure 2). Additionally, flow cytometry analysis revealed a higher percentage of Alexa 488 fluorescent cells in the SK-BR3 cell populations (71.5%) than in the MDA-MB231 cell populations (15.9%) (Figure 3b,d). Both cell populations added with untagged PCSN probe and the dye had almost no Alexa 488 fluorescent signals (Figure 3a,c). TEM micrographs revealed small electron-dense granules localized on the surface of the cell membrane of SK-BR3 cells (Figure 4). The results of an energy-dispersive spectroscopy (EDS) analysis showed a Si peak, which suggested that these granules were made of silicon.

#### 2.1.2. Imaging of HER2-Overexpressing Cells Using Dual-Imaging Probes

**Figure 5:**
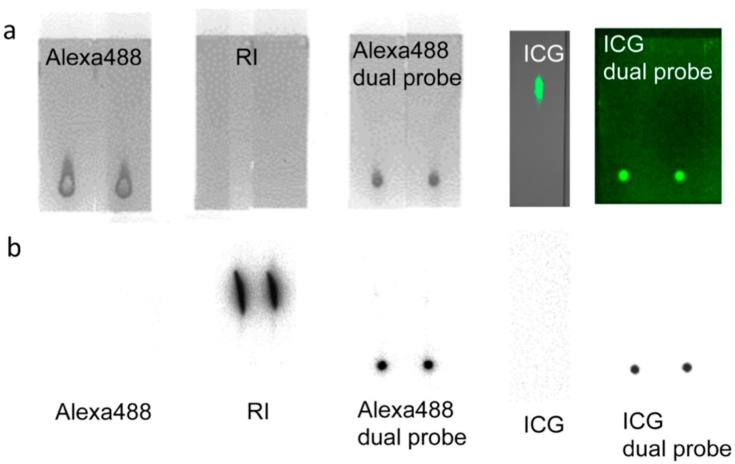
TLC analysis of dual-imaging probe. Two microliter spots of the modified PCSNs were visualized on TLC plates using fluorescence imaging (**a**) and contact radiography (**b**). Verification of complete modification was confirmed by a lack of separated spots in both studies. Combined labeling with both ^99m^Tc and Alexa 488, and both ^99m^Tc and ICG-sulfo-OSu for targeted PCSNs, were verified by TLC before each experiment. TLC was also carried out for standards (Free Alexa 488, ^99m^Tc, and ICG). They showed separation of fluorescent signals and radioactivity from spot areas (**a**,**b**).

**Figure 6:**
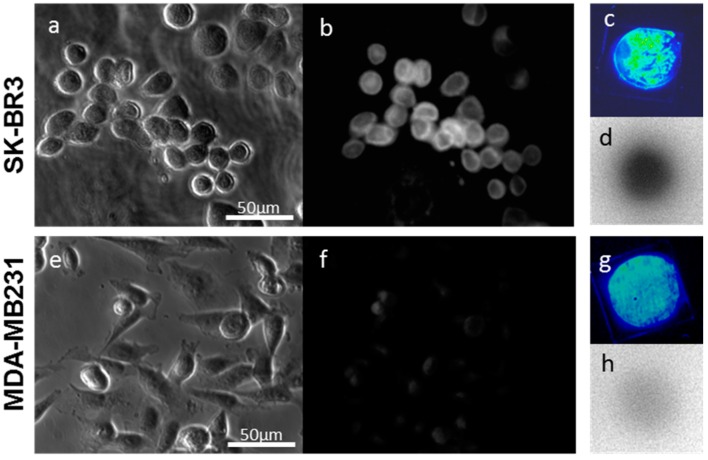
Tumor cell image of dual-imaging probe. SK-BR3 cells (**a**–**d**) show stronger near-infrared (NIR) fluorescent signals than do MDA-MB231 cells (**e**–**h**). The increased radioactivity of SK-BR3 cells (**d**) was also confirmed by phosphor imaging. The radioactivity of MDA-MB231 cells is weak (**h**). Cells are shown by phase contrast microscopy (**a**,**e**).

TLC studies revealed that the dual-imaging probes were radioactive and fluorescent but showed no separated fluorescent dyes or ^99m^Tc from the spot of dual-imaging probe. Near-infrared (NIR) fluorescence imaging of the TLC plate-processed PCSN probes modified with ICG also showed successful labeling of the probes, using the NIR imager and FLA-2000 (Figure 5a,b). TLC was also carried out for standards (Free Alexa 488, ^99m^Tc, and ICG). They showed separation of fluorescent signals and radioactivity from spot areas. Based on observations made using fluorescence microscopy, the SK-BR3 cells had stronger NIR fluorescent signals than the MDA-MB231 cells; additionally, the prominent radioactivity of the SK-BR3 cells was confirmed by phosphor imaging (Figure 6).

### 2.2. In Vivo

**Figure 7:**
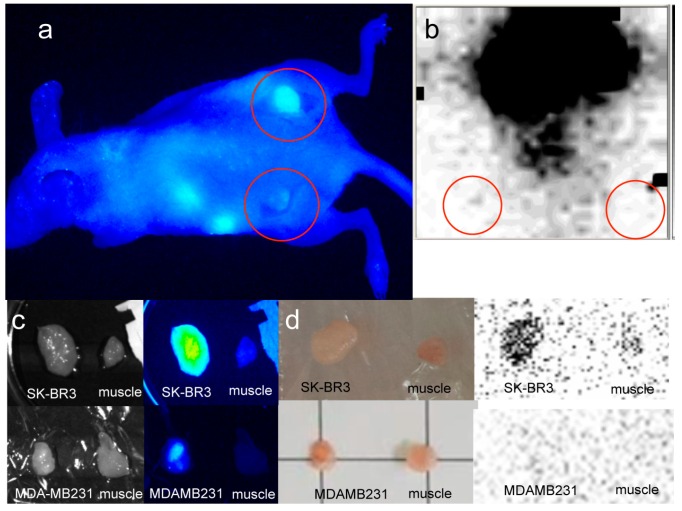

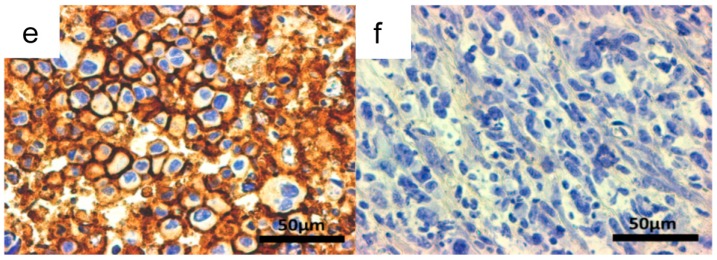
Xenografted tumors image with direct-^99m^Tc-chelated dual-imaging probe (red circles). A NIR imager reveals high fluorescence intensity in the SK-BR3 tumor (**right** dorsum, **a**) in contrast to the MDA-MB231 tumor (**left** dorsum, **a**) in a single mouse bearing both types of tumors in the direct-^99m^Tc-chelated dual-imaging probe studies. The NIR image was taken after skin incision over the xenografted tumors. The tumor-to-muscle ratios of fluorescence intensity and radioactivity were higher in the SK-BR3 tumor than in the MDA-MB231 tumor. Specific tumor uptake is not confirmed by a small γ camera with acquisition time 30 s in the lower-body image (**b**). Excised SK-BR3 tumors exhibit a higher fluorescence intensity in a NIR imager image (**c**, **upper right**) and a higher radioactivity in an FLA-2000 image (**d**, **upper right**) than do muscle tissue and MDA-MB231 tumors (**c**, **bottom right**, **d**, **bottom right**). The size of the SK-BR3 tumor is 13 mm × 8 mm × 2 mm and the size of the MDA-MB231 tumor is 6 mm × 8 mm × 5 mm (**c**,**d**). HER2 immunohistochemistry reveals stronger positive stains in the SK-BR3 tumor cells (**e**) than in the MDA-MB231 tumor cells (**f**).

**Figure 8:**
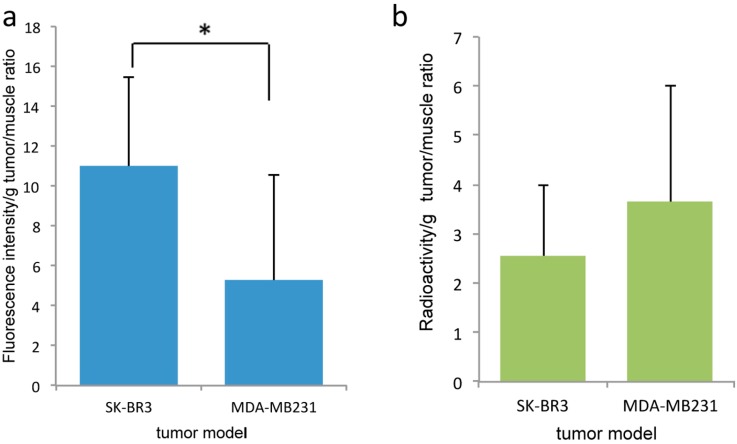
Comparison of fluorescence intensity and radioactivity in xenografted tumors on direct-^99m^Tc-chelated dual-imaging probe studies. There is a significant difference in fluorescence intensity between SK-BR3 tumors and MDA-MB231 tumors (SK-BR3, *n* = 8, MDA-MB231, *n* = 7) (**a**). However, there is no significant difference in radioactivity between SK-BR3 tumors and MDA-MB231 tumors (**b**) (* *p* < 0.05).

In the mouse tumor xenograft studies, direct-^99m^Tc-chelated dual-imaging probes and diethylenetriaminepentaacetic acid (DTPA) used ^99m^Tc-label dual-imaging probes were compared. Animal experiments with mercaptoacetyltriglycine (MAG3) used ^99m^Tc-label dual-imaging probes were not performed because of insufficient labeling of ^99m^Tc in TLC studies. In the direct-^99m^Tc-chelated dual-imaging probe studies, the NIR imager revealed a higher fluorescence intensity in SK-BR3 tumors than in MDA-MB231 tumors. In images of the removed tumor (The weight (mean ± SD) of SK-BR3 (*n* = 8) was 0.070 ± 0.041 g, MDA-MB231 (*n* = 7) was 0.076 ± 0.046 g) and muscle tissue (obtained using the NIR imager and FLA-2000), a higher fluorescence intensity and higher radioactivity were observed in SK-BR3 tumors than in muscle tissue or MDA-MB231 tumors. The single mouse bearing both types of tumors exemplified the difference between SK-BR3 and MDA-MB231; in this mouse, the SK-BR3 tumor had a higher fluorescence intensity and higher radioactivity than the MDA-MB231 tumor (Figure 7). However, analysis via a Student’s *t*-test (SK-BR3, *n* = 8, MDA-MB231, *n* = 7) indicated that there was no significant difference in radioactivity between the SK-BR3 tumor (tumor-to-muscle ratio, 2.33 ± 1.67) and the MDA-MB231 tumor (tumor-to-muscle ratio, 3.70 ± 2.30) even when the fluorescence intensity differed significantly, (tumor-to-muscle ratio, 10.64 ± 5.15) and (tumor-to-muscle ratio, 5.28 ± 5.26), respectively (*p* < 0.05) (Figure 8). HER2 immunohistochemistry reveals stronger positive stains in the SK-BR3 tumor cells than in the MDA-MB231 tumor cells (Figure 7e,f).

**Figure 9:**
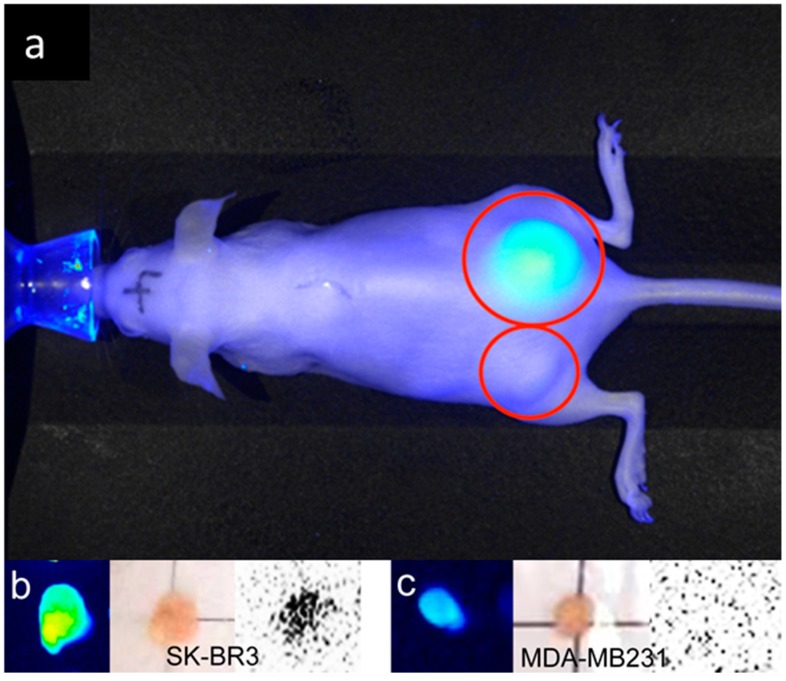
Xenografted tumors image with ^99m^Tc-diethylenetriamine pentaacetic acid (DTPA) dual-imaging probe (red circles). In vivo imaging of tumor xenograft mice with use of DTPA used dual-imaging probe. The NIR image showed a high-intensity fluorescence in the SK-BR3 tumor (**right dorsum**, **a**) in contrast to the MDA-MB231 tumor (**left dorsum**, **a**). Excised SK-BR3 tumors (**b**) exhibit a higher fluorescence intensity and radioactivity in contrast to the MDA-MB231 tumor (**c**). The size of the SK-BR3 tumor is 11 mm × 8 mm × 2 mm and the size of the MDA-MB231 tumor is 6 mm × 5 mm × 5 mm (**b**,**c**).

**Figure 10:**
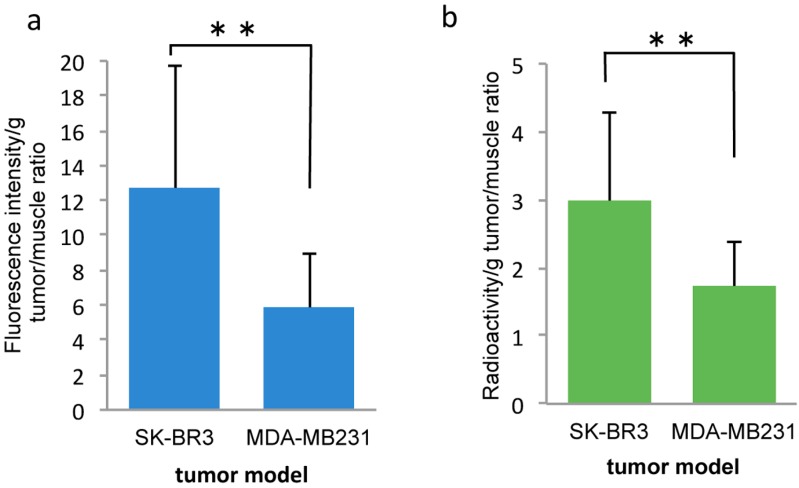
Comparison of fluorescence intensity and radioactivity in xenografted tumors on ^99m^Tc-DTPA dual-imaging probe studies. There were significant differences in fluorescence intensity and radioactivity between SK-BR3 tumors and MDA-MB231 tumors (SK-BR3, *n* = 13, MDA-MB231, *n* = 12) (**a**,**b**) (** *p* < 0.01).

**Figure 11:**
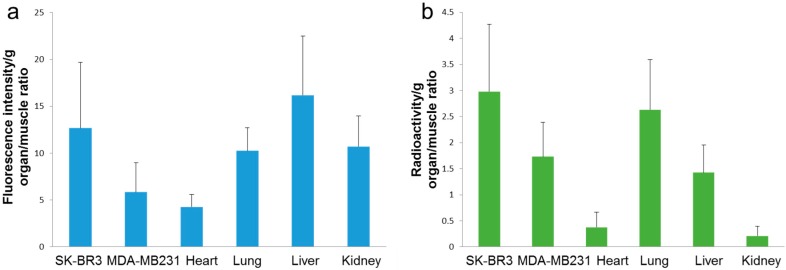
(**a**) Fluorescence intensity from the individual organs of the mice on ^99m^Tc-DTPA dual-imaging probe studies; (**b**) Radioactivity from the individual organs of the mice on ^99m^Tc-DTPA dual-imaging probe studies.

In the DTPA used ^99m^Tc-label dual-imaging probe studies, the removed tumor (The weight (mean ± SD) of SK-BR3 (*n* = 13) was 0.133 ± 0.105 g, MDA-MB231 (*n* = 12) was 0.092 ± 0.303 g), a higher-intensity of NIR was also observed in SK-BR3 tumors (tumor-to-muscle ratio, 12.68 ± 7.01) than in MDA-MB231 tumors (tumor-to-muscle ratio, 5.815 ± 3.12) (*p* < 0.01). The tumor radioactivity is higher in SK-BR3 tumors (tumor-to-muscle ratio, 2.98 ± 1.29) than in MDA-MB231 tumors (tumor-to-muscle ratio, 1.73 ± 0.66) (*p* < 0.01) in contrast to the results of direct ^99m^Tc-chelated dual-imaging probes (Figure 9 and Figure 10). The biodistribution of radioactivity and intensity of NIR in the heart, lung, liver, kidney and muscle were examined in six out of 13 mice. The fluorescence intensity and radioactivity in the liver and lung were high. However, there is a discrepancy between fluorescence intensity and radioactivity in the kidney (Figure 11).

## 3. Discussion

The dual-imaging probes were able to successfully target and image HER2-expressing cells, as confirmed by radioactivity and fluorescence measurements in cell culture experiments. The PCSNs are surface-modified functionalized silica particles. Silica nanomaterials are suitable for nanomedicine because of their high tunability, uniform production, and relatively chemically inert, biocompatible, and water dispersible properties [15,16]. Among inorganic nanoparticles, silica nanoparticles are attracting great interest for clinical applications in molecular imaging and drug delivery, although clinical trials should be carried out to demonstrate their efficiencies and safety [17,18,19]. Silica nanoparticles are categorized into two groups; solid silica nanoparticles and mesoporous silica nanoparticles. Functionalized solid silica nanoparticles have been extensively studied for use in optical imaging and drug delivery. As optical contrast agents, these nanoparticles have attractive features such as photophysical stability, biocompatibility, and favorable colloidal properties. A large exterior surface of nanoparticles can be modified with various functional substances such as antibodies, aptamers, and polymers [15]. The other group, the mesoporous silica nanoparticles, has increasingly attracted interest among researchers and clinicians not only because of the vast capacity of these nanoparticles as carriers, engendered by their unique porous structures and large surface areas, but also because of their therapeutic use in applications such as photodynamic therapy, multidrug therapy, and gene delivery [15,20]. These silica nanoparticles are part of an attractive approach for multimodality imaging. Furthermore, modification with organic materials provides an ideal nanoplatform for the assembly of multimodal nanoparticles [21]. The first clinical trial of modified fluorescent core-shell silica nanoparticles is underway and aims to create an integrin-targeting dual-modality imaging for melanoma by combining ^124^I positron emission tomography imaging with Cy5 fluorescence imaging [22,23,24].

We used an amorphous, fumed hydrophilic silica to make the dual-imaging probe. It is well known that inhalation of crystalline silica particles can cause silicosis, bronchitis, or cancer. Although crystalline and amorphous silica have the same molecular formula (SiO_2_), the structural arrangements differ, and they exhibit different biological behaviors [25]. Fumed silica or colloidal silica is a common food additive and ingredient in paints, cosmetics, and pharmaceuticals. As described previously, silica nanoparticles are considered to be biocompatible in contrast to other inorganic nanoparticles, such as quantum dots, which contain cadmium telluride crystals. However, there are still concerns regarding phagocytosis by macrophages [25]. Nevertheless, the cytotoxicity induced by nanoscale silica particles is complex and depends on the particle’s size, the surface functionalization, and the cell line used in the toxicity study [26].

The silica nanoparticles used in this study are of the solid type, and their large exterior surface is coated with PAMAM loaded with ^99m^Tc, ICG, and antibodies to target and image HER2-expressing tumor cells. The mean diameter of the dual-imaging probe was 60–80 nm. Particles or substances smaller than approximately 400 nm in diameter are likely to specifically accumulate in solid cancer tissues owing to the enhanced permeability and retention (EPR) effect. This passive targeting is generated by abnormally organized vasculatures, leakage, and impaired lymph flows [27,28,29].

Researchers have attempted to use PAMAM dendrimers as carriers for drug delivery in a variety of therapeutic applications [30]. In this study, PAMAM (generation 3) covered an average of 55.6% of the surface of particles at a thickness of approximately 3.6 nm. The NH_2_ terminal of the PAMAM was used for covalent bonding of PCSN surfaces and ICG with simultaneous ^99m^Tc labeling via chelation. 1-Ethyl-3-(3-dimethylaminopropyl) carbodiimide was employed as a carboxyl activating agent for the coupling between the superficial amines of PAMAM dendrimers and the carboxyl termini of antibodies. This simple conjugation method has the capacity to create almost any type of target needed via selection by antibodies. Furthermore, our dual-imaging probes may serve as a delivery vehicle for drugs and ligand molecules by acting as a targeted imaging carrier; this capacity is attributable to drug–PAMAM interactions, electrostatic attraction between PAMAM-amine surface groups and carboxylate groups in drug molecules, hydrophobic interactions between dendrimer cavities and drugs, and hydrogen bonds between tertiary amines within the PAMAM core and drug molecules [31]. The flexibility of dendrimers, along with their size, generation, charge, and nanoparticle hydrophilicity, influences their imaging potential by affecting their biodistribution and pharmacokinetics [32]. It is assumed that because our dual-imaging probes comprise both a silica core and a surface covered with dendrimers, they exhibit stable biological characteristics because of the low flexibility of their PAMAM dendrimers [6].

We tested the targeted dual-imaging capability (i.e., radioactivity and NIR fluorescence) of dual-imaging probes. This combined imaging system offers whole-body images that take advantage of the deeper penetration of γ rays and intra-operative NIR fluorescence images; the system facilitates surgical procedures. In this study, strong ICG fluorescent signals were observed on the cell surface of SK-BR3 cells in contrast to weak signals from the MDA-MB231 cells. These differences were also found when using other dyes, such as Alexa 555 and Alexa 488, conjugated to PCSNs. The difference was also verified by flow cytometry analysis. In cell culture experiments with contact-radiography, as well as in the fluorescence studies, radioactivity was higher in SK-BR3 cells than in MDA-MB231 cells. These results indicate that the dual-imaging probes specifically target HER2 expressing cells. In mouse experiments, SK-BR3 xenografts exhibited higher ICG fluorescent signals than did MDA-MB231 xenografts. However, in direct-^99m^Tc-chelated dual-imaging probe studies, there were no significant differences in radioactivity measurements. In the only mouse in which both cell lines were successfully xenografted, higher ^99m^Tc accumulation and higher NIR fluorescence intensity were revealed in the SK-BR3 cells than in the MDA-MB231 cells (Figure 7). The decreased accumulation of ^99m^Tc at the target sites might have occurred because of deterioration of the dual-imaging probes in the blood stream or through liver metabolism as a result of a weak chelation bond between ^99m^Tc metal ion labels and the probes. We used stannous chloride (SnCl_2_) to prepare ^99m^Tc labels. DTPA and MAG3 have been used for chelation of ^99m^Tc [33,34,35]. We expected that the DTPA and MAG3 would be coupled to silica nanoparticles by utilization of amide bond between carboxylic group from the chelator (DTPA or MAG3) and the amine group from PAMAM. DTPA was chosen to improve the instability of the ^99m^Tc labeling of dual-imaging probes in additional animal studies. MAG3 used dual-imaging probes did not show sufficient ^99m^Tc labeling in TLC studies. MAG3 is commonly used to complex ^99m^Tc [36,37,38]. However, it did not work efficiently. Unfavorable spatial distribution of the binding site on PAMAM dendrimers to MAG3 not to DTPA may render these ineffectual results because the higher generations of PAMAM dendrimer, the greater loss of conformational flexibility of the outer surfaces of the dendrimer [39]. ^99m^Tc uptake in SK-BR3 tumors was improved and significantly higher than MDA-MB231 tumors when ^99m^Tc-DTPA dual-imaging probes were used. Higher ICG fluorescent signals were also more prominent in SK-BR3 tumors than MDA-MB231 tumors. The higher radioactivity in the target tumors is expected to clearly image the target tissues from outside the body. Further studies will be necessary to make this approach more efficient for multi-modal imaging targeted at cancers.

## 4. Materials and Methods

### 4.1. Fluorescent Probes

Hyper-branched polyamidoamine (PAMAM) (generation 3) were grafted onto the surface of synthetic amorphous silica nanoparticles (Aerosil 200, Nippon Aerosil, Tokyo, Japan), as previously reported [6]. The percentage of grafted PAMAM was 55.6%. Forty mg of the processed nanoparticles (PCSNs) were dissolved in 40 mL of 50% ethanol/distilled-deionized water, mixed using an ultrasonic water bath for 30 min and centrifuged (11,000 rpm for 5 min) in spin columns (Ultrafree centrifugal filter, Durapore PVDF 0.1 µm, Millipore, Billerica, MA, USA) for purification. The concentration of silica in the purified solution was 156.69 mg/L. PCSNs were conjugated to a fluorescent dye at 37 °C for 30 min. The fluorescent dye used in this study was composed of Alexa Fluor^®^ 488, 555 carboxylicacid, succinimidyl ester, (Thermo Scientific, Waltham, MA, USA), or ICG-sulfo-OSu 2-[7-[1,3-dihydro-1,1-dimethyl-3-(4-sulfobutyl)-2H-benzo [e] indol-2-ylidene]-1,3,5-heptatrienyl]-1,1-dimethyl-3-[5-(3-sulfosuccinimidyl) oxycarbonylpentyl]-1*H*-benzo[e] indolium, innersalt, and sodium salt (Dojindo Laboratories, Kumamoto, Japan) dissolved in 1 mL of DMSO (Thermo Scientific, Hudson, NH, USA). The Alexa Fluor^®^ dyes were diluted 50-fold with PCSNs, and ICG-sulfo-OSu was diluted 100-fold with PCSNs. The fluorescence of the labeled PCSNs was verified with thin-layer chromatography (TLC) by utilizing TLC silica gel 60 F_254_ (Merck KGaA, Darmstadt, Germany), 50% ethanol-solution and a phosphor imager (FLA-2000, FUJIFILM Co., Tokyo, Japan) or a NIR imager, LI-COR Pearl Imager (700 nm channel, LI-COR Biosciences, Lincoln, NE, USA). Ten microliters of anti-HER2 antibodies (HER2/ErbB2 (D8F12) XP Rabbit mAb, (Cell Signaling Technology, Dancers, MA, USA)) and 0.8 mg of coupling reagent, 1-ethyl-3-(3-dimethylaminopropyl) carbodiimide hydrochloride, dissolved in 80 µL reaction buffer (following manufactures instructions, Peptide Coating Kit, TaKaRa, Shiga, Japan) were added to 200 µL of the fluorescently labeled PCSNs and incubated for 30 min at 37 °C. The carbodiimide reaction was used to couple PAMAM amines and the carboxyl-terminal regions of antibodies by utilizing the kit.

### 4.2. Dual-Imaging Probes

**Figure 12:**
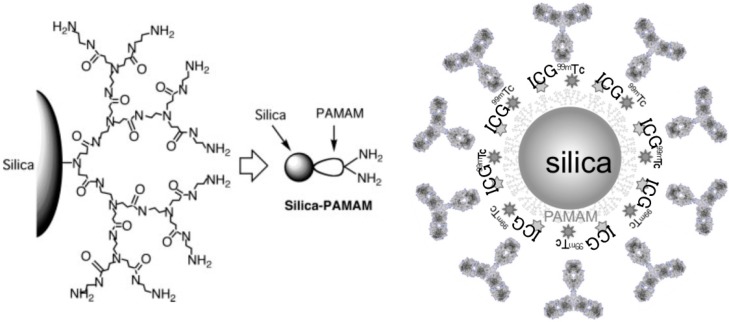
Dual-imaging probe (dual-labeled near-infrared/^99m^Tc imaging probe using PAMAM-coated silica nanoparticle).

In total, 192 µL ICG labeled fluorescent probes was conjugated to 38 µL of ^99m^Tc-solution (240 µL ^99m^Tc, (37 MBq/100 µL) added with 1 µL of Sn-solution). The Sn-solution (as a reducing agent) was composed of 20 mg of SnCl_2_ (Tin(II) Chloride, Anhydrous, 99.9%, Wako, Osaka, Japan) dissolved in 1 mL of 100% ethanol. The solutions were incubated at 37 °C for 30 min. Before the experiments were carried out, a correlation between the radioactivity and fluorescence of the labeled PCSNs was verified by TLC, a NIR imager and a phosphor imager FLA-2000. ^99m^Tc was labeled to PAMAM amine surface in an attempt by direct chelation for the dual-imaging probe. The silica and ICG concentrations in the dual-imaging probe solution were 54.5 mg/L and 7.33 micro-mol/L, respectively (Figure 12).

Chelating agents, diethylenetriaminepentaacetic acid (DTPA) and mercaptoacetyltriglycine (MAG3) were also used in this labeling for comparison.

Four hundred microliters of ICG labeled fluorescent probes were added to 160 µL coupling reagent and 15 µL DTPA-solution which was composed of 0.3 mg DTPA (Techne DTPA Kit, FUJIFILM RI Pharma Co., Tokyo, Japan) or MAG3 (Techne MAG3 injection, FUJIFILM RI Pharma Co., Tokyo, Japan) dissolved in 0.5 mL distilled-deionized water, and were incubated at 37 °C for 30 min. After that, 15 µL ^99m^Tc was added the ICG labeled fluorescent probes with DTPA or MAG3. The silica and ICG concentrations in the dual-imaging probe (DTPA) solution were 57.76 mg/L and 2.41 µmol/L, respectively. Those concentrations in the dual-imaging probe (MAG3) solution were as well as concentrations in the direct-^99m^Tc-chelated dual-imaging probe solution. Before the dual-imaging probe was used, ^99m^Tc and ICG labels were verified by TLC with a NIR imager and a FLA-2000 as well.

### 4.3. Cell Culture

The human breast cancer cell lines SK-BR3 and MDA-MB231 were obtained from the American Type Culture Collection (ATCC^®^ number HTB-30™, number HTB-26™). These cell lines were used for comparison of HER2 expression [40,41]. The HER2-positive cell line, SK-BR3 was cultured in McCoy’s5A medium (GIBCO^®^, Life Technologies, Waltham, MA, USA) supplemented with 10% fetal bovine serum (FBS, GIBCO^®^, Life Technologies, Waltham, MA, USA) and 1% penicillin-streptomycin (Invitrogen, Life Technologies, Waltham, MA, USA). Cells from the HER2-negative cell line, MDA-MB231 were cultured in DMEM medium (GIBCO^®^, Life Technologies, Waltham, MA USA) supplemented with 10% FBS and 1% penicillin-streptomycin (Invitrogen, Life Technologies, Waltham, MA, USA). Both cell lines were maintained in a humidified environment containing 5% CO_2_ at 37 °C. The medium was changed every other day.

### 4.4. In Vitro, Imaging of HER2-Overexpressing Cells Using Fluorescent Probes

In total, 2 × 10^5^ HER2-positive breast carcinoma cells (SK-BR3) and negative-HER2-expressing breast carcinoma cells (MDA-MB231) were seeded on coverslips (13 mm in diameter) at the bottoms of wells in a 24 well plate and were fixed with 4% paraformaldehyde for 15 min at room temperature. One hundred microliters of fluorescent probe was added to the cells, which were incubated overnight at 4 °C. After washing the coverslips twice with phosphate buffered saline (PBS), the samples were examined using fluorescence microscopy (Axiovert25, ZEISS, Oberkochen, Germany) with fluorescent filter (fs38HE: Excitation (Ex.) 470/40 nm Emission (Em.) 525/50 nm, fs43HE: Ex. 550/25 nm Em. 605/70 nm, ICG: Ex. 769/41 nm Em. 832/37 nm).

### 4.5. Flow Cytometry

After detaching both types of the cells with trypsin/ethylenediaminetetraacetic acid (EDTA), the cells were fixed with 1% paraformaldehyde for 15 min at room temperature. The cell suspension was washed with PBS twice. One hundred and twenty microliters of fluorescent probes was added to 1 mL of the cell suspension, which included 5 × 10^5^ cells. The cell suspension was incubated overnight at 4 °C. After washing the cell suspension with PBS twice, it was examined for fluorescence intensity (FACS Vantage SE, Becton Dickinson, Franklin Lakes, NJ, USA).

### 4.6. Transmission Electron Microscopy (TEM)

In total, 2 × 10^5^ SK-BR3 cells and MDA-MB231 cells were seeded on cover slips. They were placed in a 4% paraformaldehyde, 2% glutaraldehyde fixative in 0.1 M phosphate buffer (PB, pH 7.4) for 2 h at room temperature. They were rinsed with 0.1 M PB for 5 min at room temperature three times. During rinsing, the coverslips were cut into small pieces (3 × 3 mm). After rinsing, they were post-fixed for 1 h in 1% osmium tetroxide at room temperature and were quickly rinsed twice in distilled water. They were embedded in epoxy resin after dehydration through graded alcohols. Semithin sections were cut, stained with toluidine blue and examined under a light microscope. Ultrathin sections were cut with a diamond knife (Diatome, Biel, Switzerland) on an ultramicrotome (Reichert Jung, Vienna, Austria) and stained with platinum blue (TI blue, Nisshin EM Co., Tokyo, Japan) uranyl acetate and lead citrate. The sections were examined using a transmission electron microscope (JEM-1010, JEOL, Tokyo, Japan).

### 4.7. In Vitro, Imaging of HER2-Overexpressing Cells Using Dual-Imaging Probes

To prepare the cells for the dual-imaging probes, 2 × 10^5^ SK-BR3 cells and MDA-MB231 cells were seeded on coverslips that were place at the bottom of a 24-well plate. After 24 h, the cover slips were fixed with 4% paraformaldehyde solution. Next, 100 µL of dual-imaging probe was added to the cells and incubated for 4 h at room temperature. The cells were washed with PBS twice and were examined for fluorescence and radioactivity using fluorescence microscopy (Pearl Imager, 700 nm channel) and autoradiography with phosphor imaging plates, (IPs).

### 4.8. In Vivo, Tumor Model and Imaging of HER2-Overexpressing Cells Using Dual-Imaging Probes

Five-week-old female athymic mice (BALB-c-nu and SHO, Charles River Breeding Laboratories, Wilmington, MA, USA, *n* = 14, body weight 14–22 g) were purchased. The mice were acclimatized for 1 week and housed under conventional conditions with a 12 h light dark cycle in cages covered with filter cap; they were given free access to feed (D10001, AIN-76A, Research Diet Inc., New Brunswick, NJ, USA) and sterilized water before the experiments started.

An SK-BR3 model (*n* = 8) of breast cancer was established in mice by injecting 1 × 10^7^ cells suspended in 200 µL of a 1:1 mixture of Matrigel (Becton Dickinson, Tokyo, Japan) and PBS subcutaneously into the right dorsum. An MDA-MB231 model (*n* = 7) of breast cancer was established by injecting 1 × 10^6^ cells suspended in 200 µL PBS subcutaneously into the left dorsum of the mice. The experiments were performed 4–6 weeks after the injection.

Probes were injected into the tail vein with a 100 µL (5.9 MBq) of dual-imaging probe solution. After 4 h, the mice were observed with a small cadmium telluride (CdTe) γ camera (SSGC) and a NIR imager. Scintigraphy was performed in 14 mice. The head of the SSGC consisted of a pixelized-CdTe module (32 × 32 individual elements, total of 1024 pixels) attached to a collimator (Acrorad, Tokyo, Japan). The field of view was 44.8 × 44.8 mm [42,43]. Uptake of radioactivity was imaged 4 h after tail vein injection of dual-imaging probes under isoflurane inhalation anesthesia (Gas anesthesia system for small animals, DS pharma biomedical, Osaka, Japan with Forane^®^, Abbvie, Tokyo, Japan). After completion of the scintigraphy, the mice were imaged on a NIR imager for NIR whole animal image. Then, mice were euthanized with overdose of inhalation of isoflurane and tissues (tumor, heart, lung, liver, kidney, and muscle) were removed. The specimens were observed with FLA-2000 and a NIR imager. The excised specimens were weighed and placed on the IP. The samples were exposed on the IP in a darkroom for 60 min to produce autoradiographic images of the radioactivity, and the IP was then processed to image the radioactivity using the FLA-2000 device. Radioactivity in the specimens of ^99m^Tc was assayed using an autowell γ system (ARC-370 M, Aloka, Tokyo, Japan). Radioactivity was corrected for decay time and weight, and it is expressed as tumor-to-muscle ratios of the activity.

### 4.9. Immunohistochemistry

Excised tumors were fixed in 10% formaldehyde solution overnight and were embedded paraffin block. They were sectioned at 5 µm thickness. After deparaffinization, HER2 immunohistochemistry was performed according to a protocol of Histofine^®^ HER2 kit mono atlas (Nichirei Bioscience, Tokyo, Japan) properly with calibrated positive control and negative control.

All animals were treated in accordance with the Ethical Guidelines for Investigations of Experimental Animals of the Nippon Dental University School of Life Dentistry at Niigata (No. NDU-N-2014-123).

## 5. Conclusions

We developed functionalized silica nanoparticles loaded with ^99m^Tc and fluorescent dyes, (including ICG), for the targeting and imaging of HER2-expressing cells. The dual-modality probes efficiently imaged HER2-overexpressing cells. Although further studies are needed to produce more efficient isotope-labeling in animal studies, the results suggest that these multifunctional silica nanoparticles are a promising vehicle for imaging specific components of the cell membrane in a dual-modality manner. Furthermore, the functional capability of the nanoparticles may provide useful theranostics for delivering antitumor agents or β-emitters.
